# Proteins*Plus*: a publicly available resource for protein structure mining

**DOI:** 10.1093/nar/gkaf377

**Published:** 2025-05-06

**Authors:** Christiane Ehrt, Thorben Schulze, Joel Graef, Konrad Diedrich, Jonathan Pletzer-Zelgert, Matthias Rarey

**Affiliations:** University of Hamburg, ZBH - Center for Bioinformatics, Albert-Einstein-Ring 8-10, 22761 Hamburg, Germany; University of Hamburg, ZBH - Center for Bioinformatics, Albert-Einstein-Ring 8-10, 22761 Hamburg, Germany; University of Hamburg, ZBH - Center for Bioinformatics, Albert-Einstein-Ring 8-10, 22761 Hamburg, Germany; University of Hamburg, ZBH - Center for Bioinformatics, Albert-Einstein-Ring 8-10, 22761 Hamburg, Germany; University of Hamburg, ZBH - Center for Bioinformatics, Albert-Einstein-Ring 8-10, 22761 Hamburg, Germany; University of Hamburg, ZBH - Center for Bioinformatics, Albert-Einstein-Ring 8-10, 22761 Hamburg, Germany

## Abstract

The openly accessible Proteins*Plus* web server at https://proteins.plus is a unique resource enabling protein structure mining and modeling, focussing on protein-ligand interactions. Since its launch in 2017, the number of tools is steadily increasing. Currently, the server comprises six methods for protein structure analysis, four tools for mining the Protein Data Bank (PDB), and five prediction approaches regarding protein-ligand complex modeling. Users can use experimental structures from the PDB or computationally predicted structures from the AlphaFold Protein Structure Database as starting points. Alternatively, they can upload individual structure files. Recent updates include novel methods for detecting binding sites and predicting solvent channels in crystallographic structures, as well as updates of tools for protein-ligand interaction depiction in 2D and binding site mining. Given these updates, we present a real-life application scenario that underpins the novelties and applicability of the web server’s tools for modern structure-based design projects. It also highlights the next steps for the web server, which will be redesigned using a different technology stack to improve the inter-usability of the tools, ease maintainability, and make it future-proof.

## Graphical Abstract

**Graphical Abstract:**
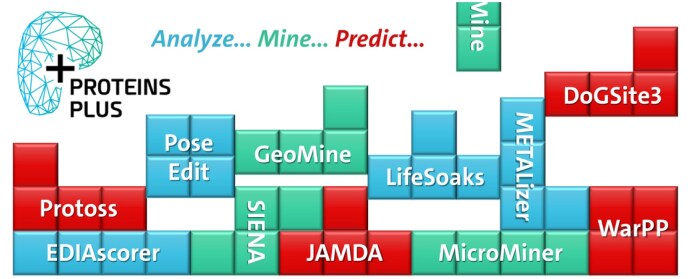

## Introduction

The Proteins*Plus* web server was established in 2017 [1] offering a selection of tools to facilitate structure-based design. It is free and open to all users without a login requirement. Eight years after its initial release, it has become a unique publicly available resource for researchers to analyze, mine, and perform predictions for protein-ligand complexes. The first analysis tool PoseView [2] for generating 2D diagrams of protein-ligand interactions was rapidly accompanied by further tools to investigate structure quality (EDIAscorer [3], StructureProfiler [4]) and metal coordination geometries (METALizer [5]). The mining tools SIENA [6], MicroMiner [7], and GeoMine [8] extended analysis functionalities towards user-friendly options for efficiently mining structures in the Protein Data Bank (PDB) [9] with tailor-made user queries through elaborate database systems. The implementation of tools to predict protein binding sites and assess their druggability (DoGSiteScorer [10]), automatically prepare protein structures for molecular docking (Protoss [11], WarPP [12]), and even perform fully automated molecular docking runs for small molecules with JAMDA [13] led from a purely analysis-focused server to an all-in-one online resource for structure-based design. Users can use PDB structures, predicted structures from the AlphaFold Protein Structure Database [14], or individual structures (with or without ligands) for analysis and prediction tasks. Thereby, the server assists in structure-based modeling studies that usually rely on applying various separate tools [15]. The availability of numerous tools in an intuitive and concise graphical user interface (see Table 1) has enabled users to instantly perform calculations that usually necessitate in-depth knowledge of various tools. It also gives rise to the wish to create pipelines and improve the interoperability of tools. Furthermore, it requires the re-examination of available tools and their ongoing development to meet the needs of the community.

**Table 1.** Proteins*Plus* tools/services – an overview

Tool	Function	Class	REST
PoseView/ PoseEdit	2D interaction diagrams	A	k
EDIA/ StructureProfiler	X-ray structure quality	A	k
LifeSoaks	X-ray solvent channels	A	
METALizer	Metal coordination geometry	A	
SIENA	Binding site similarity	M	k
MicroMiner	Single residue mutations	M	
ActivityFinder	ChEMBL compound activities	M	k
GeoMine	Geometrical site mining	M	x
Protoss	Protonation state and hydrogen atom position assignment	P	k
WarPP	Water molecule placement	P	x
DoGSite3	Binding site prediction	P	k
DoGSite- Scorer	Druggability prediction	P	k
JAMDA	Small molecule docking	P	
HyPPI	PPI classification	P	k

A: Analyze; M: Mine; P: Predict; x: REST API; k: REST API + KNIME node, the REST API enables users to programmatically access the web services by creating jobs and downloading the results using the command line

Here, we present recent updates and highlights of Proteins*Plus*. Besides method enhancements (DoGSite3 [16], PoseEdit [17], GeoMine [18]), we describe a novel tool that enables users to find and analyze solvent channels in macromolecular structures solved by X-ray crystallography and provide insights into combining various tools in the Konstanz Information Miner KNIME [19]. Finally, we will give a short outlook on the redesign of the web server and upcoming extensions of the tool portfolio.

## Materials and methods

### DoGSite3

Identifying protein-ligand binding pockets and their properties is one of the core tasks in structure-based drug design. With DoGSiteScorer [10], developed in 2010, we aimed at a precise and fully automated binding pocket prediction and druggability assessment. In 2023, we remastered the algorithm to improve the pocket property robustness, prediction accuracy, ligand, and pocket coverage. Furthermore, we added a new mode to compute ligand-biased, difficult-to-detect binding pockets covering the ligand volume with reasonable pocket boundaries. The algorithm was optimized for performance, leading to substantially improved runtimes. This new method, DoGSite3 [16], can be applied to protein and nucleic acid structures via Proteins*Plus*. Its performance in predicting small molecule binding sites is on par or even better than that of other geometry-based approaches.

DoGSite3 maps the macromolecular structure onto a 3D grid. Principal component analysis is used to standardize the orientation such that the required grid size is minimized and the pocket prediction becomes orientation-invariant. Each grid point is annotated as a macromolecule or solvent point based on its distance to the closest macromolecular atom. By calculating the Difference-of-Gaussian filter (DoG value), we can identify locations in the grid that favor spherical cavities. We also compute protein-solvent-protein events [20, 21] using a scanline procedure along all three principal axes and four cube-corner diagonals to indicate the buriedness of a grid point. The DoG values and PSP event counters are then used to cluster the grid points into pockets, which are enlarged by a dilution radius and merged into larger pockets. Finally, small pockets of (V < 20 Å^3^) are removed. Pocket descriptors are calculated for the remaining ones. The DoGSite3 parameters were optimized on two datasets of the binding site comparison benchmark ProSPECCTs [22] towards high robustness of predicted binding pocket properties.

At Proteins*Plus*, users can select or upload a structure of interest to have its pockets predicted. Users can choose a ligand to calculate the corresponding ligand and pocket coverage. In addition, the new ‘Ligand Bias’ mode can be enabled to predict ligand-biased pockets. In this case, a ligand of choice is used to annotate close grid points, increasing the probability that these grid points will become part of pockets. Predictions can also be restricted to macromolecular chains of interest.

The resulting pockets (with their key properties such as volume, surface, depth, etc.) are displayed in a table and visualized in the NGL viewer panel. Users can download the pockets in CCP4 and PDB format and a text file with the calculated pocket properties. Thus, DoGSite3 enables reliable pocket prediction, robust property calculation, and the assignment of ligand-biased difficult-to-detect pockets within seconds. It belongs to the most widely used tools available on Proteins*Plus*.

### GeoMine

Detecting binding sites with certain 3D structural features can be valuable in drug development-related tasks like drug repurposing and side effect analyses [23]. GeoMine, a 3D search engine for binding sites in the PDB, has been a part of the Proteins*Plus* server’s tool suite since 2021 [8].

To build a 3D query, users can define various chemical feature points, like atoms, and relative spatial relationships, such as distance constraints. They can directly place its components in the 3D viewer and select them from a ligand-bound or ligand-unbound 3D binding site, which is precalculated on the fly by DoGSite3 for a user-specified input structure. The 3D viewer-specified query components are subsequently loaded into tables, enabling further refinement of various properties, such as the element of an atom or the length variance of a distance constraint. The tool has undergone multiple updates since its initial release to assist users in managing the high complexity of the 3D query and its design process. The newest version, released in 2024, further improves the search interface’s usability by enabling query design in 2D [18].

For a user-specified ligand inside a precalculated ligand-bound 3D binding site, the new 2D query editor enables loading a corresponding 2D depiction of the ligand binding mode, showing the ligand, its interaction partners, and relative intermolecular interactions. 2D diagrams are generated by PoseEdit [17]. The 2D diagrams display additional 3D-visualized chemical feature types of interest, like aromatic ring centers or secondary structure points, further adapting their content to 3D-visualized binding sites.

**Figure 1.**
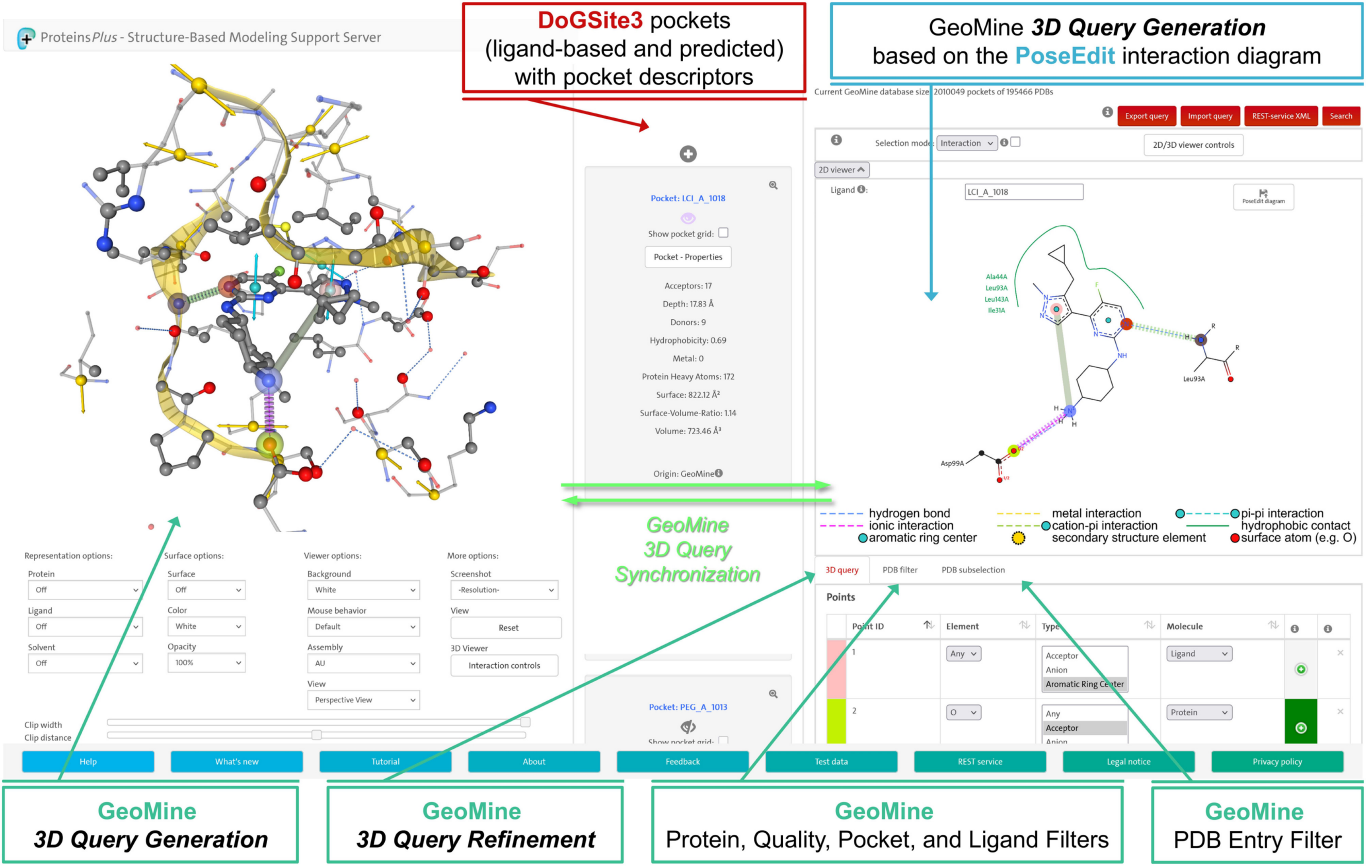
Proteins*Plus* interface depicting a GeoMine query for PDB entry 6gzd [25] with the DoGSite3-calculated pocket for the small molecule inhibitor [4-[[4-[5-(cyclopropylmethyl)-1-methyl-pyrazol-4-yl]-5-fluoranyl-pyrimidin-2-yl]amino]cyclohexyl]azanium (residue name LCI).

Consequently, users can now create a query in 2D, 3D, or simultaneously in both views, exploiting the synchronized query design and highlighting. Synergistically integrated textual, 2D, and 3D input types for query definition have their respective strengths. 3D input formulation is typically more complex and time-consuming, requiring extensive translation, rotation, and zooming of the 3D scene. However, a 3D binding site highlights spatial information and more detailed chemical information, which might be relevant for query selection. In contrast, the 2D visualization provides no spatial information. However, a 2D diagram might be superior for query design if its restricted information content is sufficient since it highlights the key aspects of a ligand binding mode. In this case, the immediate focus on crucial chemical information provided by a 2D scene enables a faster and easier query design process (see Fig. 1).

In its latest version, GeoMine significantly enhances handling the query’s complex 3D data type by providing a simplified structural template representation for query design. Comparable freely available tools such as the structure motif search for the PDB [24] are available as web services but have fewer search capabilities and query design options.

### PoseEdit

In a drug development process, visualizing the binding modes of candidate compounds via 2D graphical representations is a common task for concisely communicating, analyzing, understanding, and potentially improving their activity. Such graphical representations are commonly part of conference posters, slide presentations, or scientific articles.

The tool PoseView [2] became part of the Proteins*Plus* server to automatize 2D visualizations early on. For a user-specified protein-ligand complex, PoseView’s algorithm automatically creates a layout and depicts a 2D diagram of the corresponding ligand binding mode with atomistic detail following chemical drawing conventions. A 2D diagram illustrates the ligand, its interaction partners, and intermolecular interactions of different types. In 2023, the tool PoseEdit [17] was integrated into the Proteins*Plus* server, presenting PoseView’s drawing functionality within a significantly extended graphical user interface to further assist scientists in visualizing ligand binding modes to themselves or others.

PoseEdit’s 2D editor allows users to comprehensively explore, modify, and improve 2D diagrams, overcoming otherwise unsolvable subjective or objective diagram limitations imposed by the tool’s graphical and chemical presets or the quality of the layout algorithm’s output. For example, a user might want to address the coloring of intermolecular interactions, protonation states, or graphical collisions between chemical structures. The easy-to-use and highly functional 2D editor offers specialized modes to move, rotate, add, remove, mirror, highlight interactive diagram objects, and edit their depicted graphical and chemical properties in a chemistry-aware manner. Additional features, such as the editor’s history to undo and redo changes, the export of textual ligand binding mode information as a file, or the download and upload of 2D diagrams, further support the exploration, comprehension, and modification of 2D pose diagrams.

Furthermore, the 2D viewer is complemented by the 3D viewer, which provides the corresponding complete binding site in 3D. The 3D binding site is calculated by DoGSite3 and highlights additional relevant information, e.g. 3D spatial information and chemical information like non-interacting chemical structures or intermolecular interactions with solvent molecules. The synchronization feature of both viewers allows the simultaneous highlighting of mouse pointer-focused atoms, bonds, and chemical structures, clarifying the visual correspondence between both scenes. Consequently, users can explore both representation types to verify key aspects of a ligand binding mode synergistically and to gain ideas on improving the chemical information content of the 2D diagram.

PoseEdit also introduces an updated interaction model that applies new calculation criteria and detects additional types of intermolecular interactions. The model now includes covalent bonds and ionic interactions between the ligand and residues. It also represents cation-pi and pi-stacking interactions with greater detail by associating them with individual rings rather than entire ring systems. Furthermore, PoseEdit provides some general graphical improvements, such as depicting intermolecular interactions and bonds with color gradients and a collision-reducing minimal atom radius in which bonds and intermolecular interactions cannot extend.

Given these advances, PoseEdit offers a new way to improve the usability of all its automatically generated 2D diagrams by interactively including the user’s input.

### LifeSoaks

Structure-based design tasks are often accompanied by crystallographic screenings of compound or fragment libraries [26, 27]. While the systematic search for binders in an X-ray crystallography setting delivers valuable insights into the binding mode of potential inhibitors, these methods come with the burden of requiring hundreds to thousands of crystallization experiments. Therefore, a common approach is to soak preformed protein crystals for which established crystallization protocols exist in solutions of the molecules of interest and determine the presence of binders in subsequent diffraction experiments [28]. Since protein crystals always contain a substantial amount of solvent [29], small molecules might diffuse into them and reach the potential binding pocket. However, for an experimentalist, it is not trivial to identify whether a free path to these locations exists in the complex geometric arrangement of the crystal.

**Figure 2.**
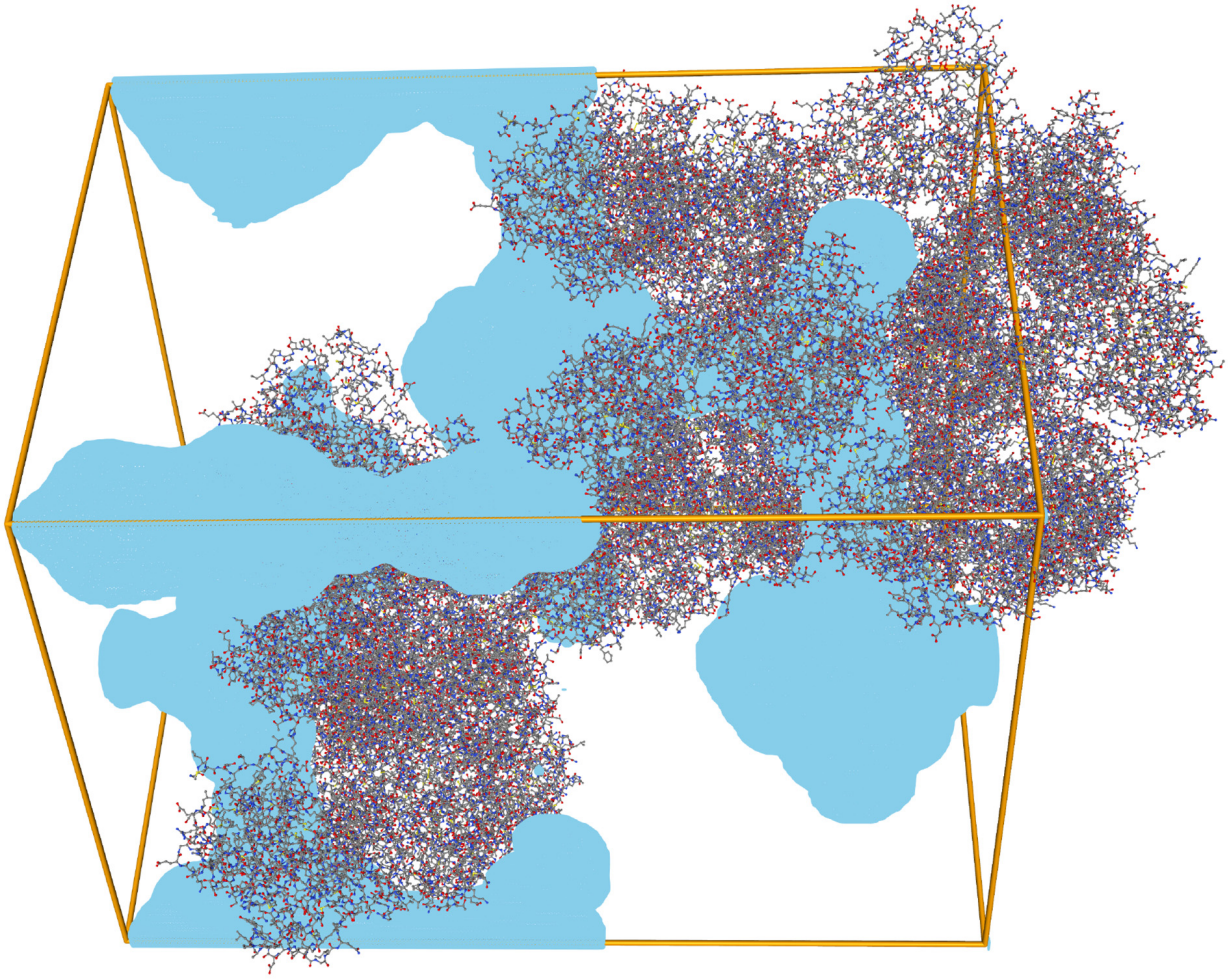
Calculated solvent channels for PDB entry 5seq [33] visualized with an 11 Å cut-off. Several cavities in the unit cell exist that are connected by channels with a radius of 11 Å. Reducing the cut-off radius would increase the amount of space visualized as a channel since a smaller spherical probe would be able to pass more narrow bottlenecks.

LifeSoaks was developed to address this issue by analyzing the periodic channel arrangements with a special focus on geometric bottlenecks [30]. The Proteins*Plus* web server contains a simplified LifeSoaks interface. Users can either start the default calculation of solvent channels without specifications or with a pocket of interest by selecting a reference ligand. In both cases, a CCP4 grid file [31] will be generated. The file represents solvent channels for one crystallographic unit cell. Each grid point is annotated with the so-called local bottleneck radius, which describes the maximum size of a spherical object that can reach that specific position on a periodic path through the crystal. This measure thereby not only represents the local vacant space but also includes bottlenecks anywhere on the path to this position. When the calculation is finished, it is recommended that the unit cell assembly option is selected for the protein when inspecting the channels. Using the radius cut-off slider, all grid points with a local bottleneck radius greater or equal to the currently selected value will be visualized as shown in Fig. 2. The overall bottleneck radius represents the largest periodic path in the structure. If the user specified a pocket, the values ‘inner/outer active site radius’ represent the bottleneck radius inside and in front of the pocket. Users can download the calculated CCP4 file for visual inspection with any molecular visualization software.

MAP_CHANNELS [32] offers similar channel predictions as a standalone tool. Compared to LifeSoaks, it suffers from very long run times for large unit cells and its unavailability as a web service. Therefore, LifeSoaks is the ideal choice for occasional users interested in finding solvent channels in protein structures.

## Application

### Binding site mining

The human casein kinase 1 isoform α (CK1α, UniProt accession: P48729) is a component of the Wnt/β-catenin signaling pathway [34]. It is a negative regulator of Wnt signaling. Its activation might be promising to treat Wnt-driven cancers [35] and neurological disorders [36]. Furthermore, CK1α inhibitors have been explored for cancer therapy [25]. Therefore, identifying potential binding sites and their similarity to known binding sites might help to understand the enzyme’s function and learn about previously unknown small molecule regulators.

Four experimental structures of CK1α are known. We start our explorations with the PDB entry 8g66 [37]. The structure is a complex of CK1α with CRL4CRBN ubiquitin ligase responsible for its degradation. Only protein chains C and F represent our target of interest. We can use DoGSite3 to predict binding sites, restricting the selection to chains C and F. Nine pockets were detected in chain C. We can add the detected pockets to the Pockets tab for further analysis. Next, we want to find related binding sites. Therefore, we use SIENA to search for related binding sites. We can use the predicted binding sites from the Pockets tab and run the SIENA calculations in the ‘flexibility analysis’ mode. It identifies all conformational and sequential variations of an experimentally observed binding site. The sequence similarity has to be at least 30%.

For the pocket P_1, we get 150 results. Upon inspecting the most similar sites in terms of backbone root mean square deviation, we learn that this site is often occupied by inorganic anions (e.g. PDB entry 4xhl [38]). The structurally most different sites bind substrate peptides (e.g. PDB entry 8d7p [39]). We learn that no similar sites are occupied by organic small molecules when sorting the result table according to the ligand ID. Repeating this analysis for pocket P_2, we find that most of the 144 similar sites bind small molecules. Inspecting the matching entries, we can quickly see that this is the ATP-binding pocket of the enzyme. Finally, we search for pockets similar to pocket P_3. However, we only find structures of CK1α. Only one hit binds an ATP-competitive binder (PDB entry 6gzd [25]). As protein conformations are variable and depend on experimental conditions and interaction partners, exploring all available structures in this way might become time-consuming and less reproducible. Therefore, the following section introduces a way to automatize such analyses.

### Combining the input and output of Proteins*Plus* Tools

**Figure 3.**
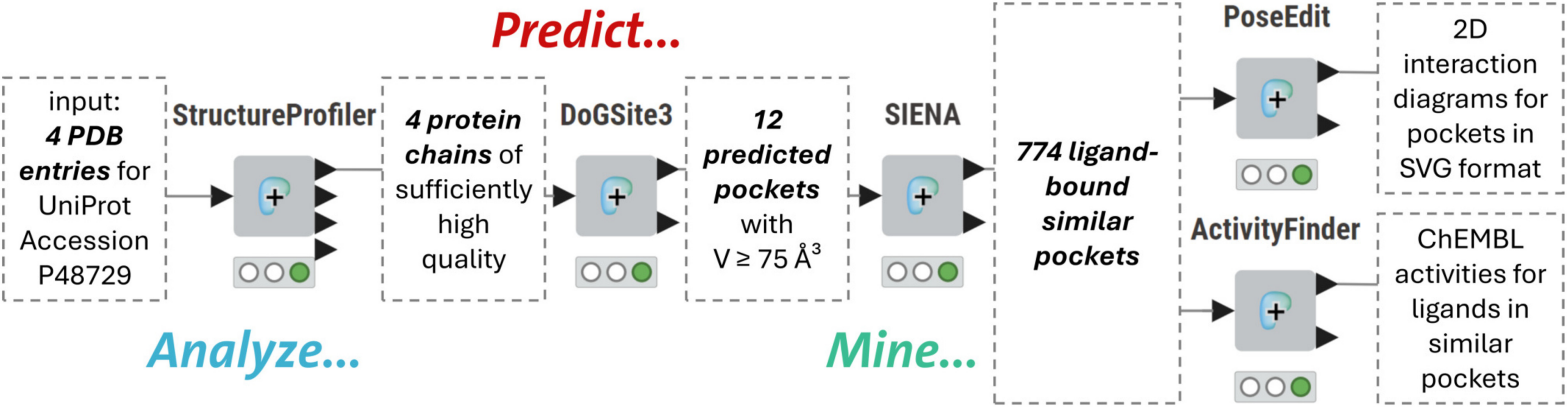
Schematic view of the fully automated KNIME workflow to explore the binding site space of human casein kinase 1 isoform α. A more detailed view of the complete workflow can be found at https://hub.knime.com/s/mA3oXwb3B8vyCA0W

API requests to several tools (see Table 1) can also be sent via the command line, enabling users to incorporate these tools in interactive data mining workflow engines such as KNIME [19]. Instead of repeating these manual analyses for all available structures and predicted pockets, we can use the Proteins*Plus* KNIME nodes to quickly explore the binding site space of our target (see Fig. 3; https://hub.knime.com/proteinsplus). Upon entering the UniProt accession for a protein of interest, the KNIME workflow automatically finds all available structures in the PDB, checks their structure quality with StructureProfiler, extracts the relevant chains for the high-quality PDB entries, and automatically starts DoGSite3 jobs on the corresponding REST service for all relevant chains. The output contains a table with calculated binding site descriptors and a list of all binding site-defining residues for the four chains. Using the descriptor information, we can, for example, extract binding site-defining residues with a volume of at least 75 Å^3^ (12 pockets). These residues and their corresponding PDB codes are forwarded to automatically generate and run SIENA queries. The resulting 888 hits can be filtered based on the presence of ligands in the matching sites. Then, we can apply the ActivityFinder node to search for experimentally determined activities of the 309 ligands. We learn that the small molecules have reported K_*i*_ and IC_50_ values and, therefore, represent inhibitors of the enzymes. Using the freely available cheminformatics nodes by CDK [40], we can also look for the most similar ligands to known CK1α inhibitors. Finally, we can automatically generate 2D interaction diagrams with PoseEdit by forwarding the PDB codes and ligand identifiers from the SIENA search to the respective node to explore the ligands’ interactions with the binding sites of related enzymes. The described KNIME workflow can be downloaded at https://hub.knime.com/s/mA3oXwb3B8vyCA0W and adjusted to any target of interest.

## Summary and outlook

In the past years, several developments on Proteins*Plus* considerably improved its applicability for not only analyzing and mining protein-ligand complexes but also crucial prediction tasks in structure-based drug design. The methodologies behind DoGSite3 and PoseEdit were successfully integrated into GeoMine. EDIAscorer and LifeSoaks assist users in investigating protein crystal structures.

In our ongoing effort to improve the Proteins*Plus*, it is currently fully redesigned with a new technology stack (Django backend [41], Vue.js frontend [42]). The consequent backend-frontend separation facilitates independent scaling and focused development, ensures improved long-term maintainability, optimizes job scheduling efficiency, and provides easy access to the extensive Python software ecosystem. A major objective of the new Proteins*Plus* architecture is intuitive interoperability among its tools. One guiding principle is that tools that naturally complement each other should seamlessly interoperate. For example, pre-computed pockets generated by GeoMine will be directly applicable to SIENA searches, streamlining user workflows. Besides, the Proteins*Plus* web server is continuously updated with new features, visualizations, and tools.

While supporting the life science community as a whole, Proteins*Plus* substantially lowers the barrier to using protein-ligand modeling approaches to occasional and first-time users, making it attractive in education. Taken together, we hope that the discussed and upcoming developments of Proteins*Plus* will further support and considerably facilitate common tasks in structure-based design.

## Data Availability

All data underlying this manuscript is available online at https://proteins.plus/ and https://hub.knime.com/proteinsplus.
